# Relationship between plasma atherogenic index and subclinical hypothyroidism: an analysis of NHANES data and animal experiment

**DOI:** 10.3389/fendo.2025.1700853

**Published:** 2025-11-03

**Authors:** Qiwei Chen, Yuwan Li, Yi Ruan, Linxi Jin, Shuhong Yao, Zhuang Han, Xinmiao Hong, Zhita Wang, Liang Li, Weidong He, Liuqing Yang, Xianpei Heng

**Affiliations:** ^1^ Department of Endocrinology, People’s Hospital Affiliated to Fujian University of Traditional Chinese Medicine, Fuzhou, Fujian, China; ^2^ First Clinical Medical College, Fujian University of Traditional Chinese Medicine, Fuzhou, Fujian, China; ^3^ Department of Geriatrics, People’s Hospital Affiliated to Fujian University of Traditional Chinese Medicine, Fuzhou, Fujian, China

**Keywords:** atherogenic index of plasma, thyroid function, subclinical hypothyroidism, NHANES, animal experiment

## Abstract

**Background:**

The relationship between subclinical hypothyroidism (SCH) and dyslipidemia is established, but that between the atherogenic index of plasma (AIP) and SCH remains unknown. This study aimed to investigate this association by combining an analysis of NHANES data with experimental evidence from an animal experiment.

**Methods:**

Cross-sectional data from 3,135 adults were analyzed. Weighted regression and linear models assessed associations between AIP (and its quartiles) and SCH and thyroid hormones. Restricted cubic splines (RCS) tested nonlinearity. Mediation analysis was utilized to identify the mediating effects of thyroid-stimulating hormone (TSH). Subgroup analyses and interaction tests were employed to explore the association between AIP and SCH. To validate these findings, a Sprague-Dawley rat model was established with a high-fat diet and the rats were divided into a control group (CG) and a model group (MG). Blood Lipid, AIP and thyroid function (TSH, FT3, FT4) were measured in each group.

**Results:**

After multivariable adjustment, the highest AIP quartile (Q4) significantly correlated with higher SCH prevalence. Elevated AIP associated with decreased free tetraiodothyronine (FT4) and increased total thyroxine (TT4), free triiodothyronine (FT3), total triiodothyronine (TT3), and TSH. RCS showed linear relationships of AIP with SCH, FT4, FT3, and TSH, but nonlinear with TT3 and TT4. Additionally, mediation analysis indicated that TSH accounted for 39.76% of the observed association between AIP and SCH. Animal experiments confirmed that compared with the CG, rats in the MG exhibited significantly higher levels of blood lipid, AIP and TSH, but lower levels of FT4 and FT3.

**Conclusion:**

Elevated AIP is significantly associated with a higher prevalence of SCH, and TSH is an interrelated factor in this association. Experimental evidence also shows a link between AIP elevation and thyroid homeostasis disruption, suggesting a relationship between AIP and thyroid dysfunction.

## 1 Introduction

Subclinical hypothyroidism (SCH), defined by elevated thyroid-stimulating hormone (TSH) with normal free thyroxine (FT4) levels, elevates the risk of cardiovascular mortality, atherosclerosis, and dyslipidemia (1–3). This link is well-established: both human and animal studies consistently show altered lipid profiles—including elevated total cholesterol (TC), low-density lipoprotein cholesterol (LDL-C), and triglycerides (TG)—in SCH (4–6), highlighting a close pathophysiological connection.

The atherogenic index of plasma (AIP), calculated as log(TG/HDL-C), offers an integrated view of lipid metabolism. It reflects the balance between pro-atherogenic and protective lipoproteins and strongly correlates with atherogenic small, dense LDL-C particles (7, 8). Due to its sensitivity to metabolic changes, AIP is considered a superior predictor of cardiovascular risk and dyslipidemia compared to conventional lipid measures (9, 10).

The relationship between dyslipidemia and SCH is likely bidirectional. While SCH promotes lipid abnormalities, dyslipidemia may also impair thyroid function through inflammation, oxidative stress, and suppressed deiodinase activity (11–13). Despite this interplay, the association between AIP and SCH remains unexplored.

Given AIP’s predictive power for dyslipidemia and the established interplay between lipid metabolism and SCH, we hypothesized an association between AIP and SCH. We utilized the National Health and Nutrition Examination Survey (NHANES) database to investigate the association between AIP and SCH, an area that has, to our knowledge, not been previously explored in a large, nationally representative population. To complement these population-based findings, we conducted an animal experiment. The primary rationale for this experimental approach was to examine the relationship between AIP and thyroid parameters under highly controlled conditions. By using a genetically homogeneous animal model and a standardized high-fat diet, we aimed to minimize the extensive confounding factors (e.g., varying medication use, diverse genetic backgrounds, and inconsistent dietary habits) that are inherent in observational human studies. This approach allows for an assessment of the AIP-thyroid function association in a more isolated physiological context, strengthening the biological plausibility of the epidemiological observations.

## 2 Materials and methods

### 2.1 Epidemiologic analysis of relationship between AIP and SCH in NHANES data

#### 2.1.1 Data sources

Our study utilized NHANES data collected between 2007 and 2012. NHANES employs a multistage, stratified probability sampling design to obtain a nationally representative sample in the United States. All participants completed detailed household interviews and underwent standardized physical examinations, aimed at assessing the health and nutritional status of the U.S. population. Data from different survey cycles were merged using the unique participant identifier — the “Sequence Number” variable.

**Figure 1:**
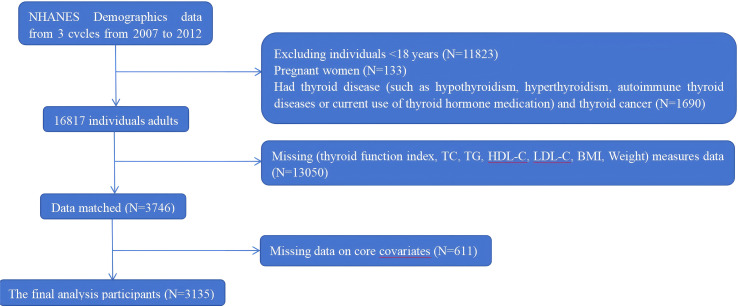
Flow chart of participant recruitment.

A total of 30,442 participants were enrolled. We excluded participants according to the following criteria: (1) aged < 18 years (n = 11,823); (2) pregnancy (n = 133); (3) history of thyroid disease (including overt hypothyroidism, hyperthyroidism, autoimmune thyroid diseases, or current use of thyroid hormone medication), or thyroid cancer (n = 1690); (4) missing data on thyroid function indices, lipid profiles (such as TC, TG, HDL-C), BMI, or other key covariates (n = 13,050); and (5) incomplete covariate information (n = 611). Therefore, a total of 3135 eligible participants were included in the final analysis. A detailed flowchart of participant selection is presented in **Figure 1**.

#### 2.1.2 Ethical considerations for NHANES data

The NHANES protocol was approved by the Institutional Review Board of the National Center for Health Statistics (NCHS), and written informed consent was obtained from all participants. Detailed information regarding the survey design, sampling methodology, and laboratory procedures is available on the NHANES website (http://www.cdc.gov/nchs/nhanes).

#### 2.1.3 Calculation of AIP levels

AIP was used as the exposure variable. AIP was calculated based on serum levels of TG and HDL-C, using the formula (14): log_10_ [TG (mmol/L)/HDL-C (mmol/L)]. The baseline characteristics of the participants were presented based on the quartiles of AIP. As established cardiovascular AIP cutoffs are not validated for thyroid outcomes (15, 16), we employed quartile-based analysis to objectively assess this association without applying unproven thresholds.

#### 2.1.4 Thyroid function assessment

In this investigation, SCH defined as a serum TSH concentration exceeding 4.5 mIU/L with concomitant FT4 levels within the euthyroid reference interval (0.6-1.6 ng/dL), was employed as a primary outcome variable (17). The study incorporated thyroid functional biomarkers as an outcome variable, including TSH, total thyroxine (TT4), total triiodothyronine (TT3), free triiodothyronine (FT3), FT4, and thyroglobulin (Tg). Laboratory assessments were conducted utilizing standardized methodologies: TT3, FT3, TT3 and Tg were quantified via competitive binding immunoassays, while FT4 was determined using a two-step enzymatic immunoassay. TSH measurements were executed through third-generation two-site immunometric assays. All biochemical determinations were sourced from the NHANES laboratory repository.

#### 2.1.5 Covariates

The covariates encompassed demographic and health-related characteristics, including gender, age, race, education, smoking status, alcohol intake, body mass index (BMI), urinary iodine concentration, diabetes, and cardiovascular disease. Participants were categorized into three age groups according to World Health Organization criteria: young adults (18–44 years), middle-aged adults (45–59 years), and older adults (≥ 60 years). Racial and ethnic groups were classified as Hispanic or other Hispanic, non-Hispanic White, non-Hispanic Black, or Other. Educational level was stratified as follows: less than 9th grade, grades 9 - 11, high school graduate, and some college. These categories approximately correspond to basic education (less than 9th grade), some secondary education (grades 9 - 11), completed secondary education (high school graduate), and some higher education without a degree (some college). Participants were categorized based on smoking history as current smokers, former smokers, or never smokers. Drinking status was classified into the following categories: non-drinker, 1–4 drinks per month, 5–9 drinks per month, and ≥10 drinks per month. BMI was calculated as weight in kilograms divided by height in meters squared. According to World Health Organization guidelines, participants were classified as follows: normal weight (18.5 < BMI ≤ 24.9 kg/m²), overweight (25 kg/m² ≤ BMI ≤ 29.9 kg/m²), or obesity (BMI ≥ 30 kg/m²). Blood samples were collected at a mobile examination center, stored at –20°C, and subsequently shipped to a central laboratory for analysis of TC, TG, LDL-C, and HDL-C using standardized procedures. Diabetes was defined based on self-reported diagnosis or current use of anti-diabetic medication. Cardiovascular disease (CAD)—including heart failure, coronary heart disease, angina, myocardial infarction, and stroke—were identified through self-reported physician diagnoses of these conditions.

### 2.2 Animal experiment validating the relationship between AIP and thyroid function

#### 2.2.1 Equipment and reagents

Microplate Reader (TECAN (Shanghai) Laboratory Equipment Co., Ltd); TC, TG, HDL-C and LDL-C kits (Nanjing Jiancheng Bioengineering Insitute (China), A110-1-1, A111-1-1, A1113-1-1, and A112-1-1, respectively); TSH, FT3 and FT4 kits (Jiangsu Meimian Industrial Co., Ltd (China), MM-0573R2, MM-20356R2, and MM-0584R2, respectively).

#### 2.2.2 Experimental animals

Twenty 7-week-old male Sprague-Dawley (SD) rats (weight: 230 ± 10 g) were purchased from Shanghai Sleik Laboratory Animal Co., Ltd. (License No. SCXK (Shanghai) 2022-0004). The animals were housed under specific pathogen-free conditions in the Laboratory Animal Center of Fujian University of Traditional Chinese Medicine (License No. SYXK (Min) 2023-0004). Environmental conditions were strictly controlled: temperature was maintained at 22 ± 2°C with daily fluctuations less than 4°C, relative humidity at 50 ± 5%, and a 12-hour light/dark cycle. Rats had ad libitum access to food and water. Bedding was replaced regularly to ensure a dry and hygienic environment. All animals were acclimatized for one week prior to experimentation. The sample size of ten rats per group was determined based on common practices in similar studies investigating high-fat diet-induced dyslipidemia in rodent models (18, 19). This sample size has been empirically demonstrated to be sufficient for detecting significant differences in key metabolic and endocrine parameters.

#### 2.2.3 Ethical considerations for animal experiment

The experimental protocol was approved by the Medical Ethics Review Board of Fujian University of Traditional Chinese Medicine (Ethics Registration No.: W2024044). All procedures were conducted in strict accordance with institutional ethical guidelines and relevant regulations for animal research to ensure the welfare of laboratory animals throughout the study.

#### 2.2.4 Establishment of the dyslipidemia model

The SD rats were randomly assigned to two groups: a control group (CG) and a model group (MG), with ten rats in each. The CG received a standard chow diet, while the MG was fed a high-sucrose and high-fat diet (composition: 70.7% standard diet, 20% sucrose, 7% refined lard, 2% cholesterol, and 0.3% sodium cholate) for 12 weeks. After the intervention, the model was considered successful, as evidenced by significantly elevated serum levels of TC, TG, and LDL, along with a decrease in HDL in the MG compared to the CG (20).

#### 2.2.5 Sample collection and biochemical assays

General anesthesia was induced via intraperitoneal injection of 20% urethane (5 mL/kg) combined with inhalation of carbon dioxide in an anesthesia chamber. Upon achieving a surgical plane of anesthesia, the abdominal cavity was opened, and blood samples were collected from the abdominal aorta. The whole blood was allowed to clot at room temperature for 30 minutes, followed by centrifugation at 3000×g for 15 min at 4°C. The serum supernatant was carefully aliquoted and stored at −80°C until analysis. Blood Lipid, AIP and thyroid function (TSH, FT3, FT4) were measured in each group. This design allowed us not only to compare group differences but also to explore the correlative relationships between AIP and thyroid parameters under different metabolic conditions. Serum levels of TC, TG, HDL-C, and LDL-C were measured using commercial enzymatic assay kits (Nanjing Jiancheng Bioengineering Insitute, China, A110-1-1, A111-1-1, A1113-1-1, and A112-1-1, respectively) following the manufacturer’s instructions. Serum concentrations of TSH, FT3, and FT4 were determined using enzyme-linked immunosorbent assays (ELISA) (Jiangsu Meimian Industrial Co., Ltd (China), MM-0573R2, MM-20356R2, and MM-0584R2, respectively). All assays were performed in strict accordance with the protocols of manufacturers.

### 2.3 Statistical analysis

All statistical analyses were performed using R software (version 4.5.1, http://www.R-project.org) and EmpowerStats (version 5.2, http://www.empowerstats.com). A two-sided P value of less than 0.05 was considered statistically significant.

To account for the complex, multi-stage sampling design of NHANES, all analyses incorporated sampling weights, stratification variables, and clustering factors. This approach ensures nationally representative estimates and prevents overestimation of statistical significance. Continuous variables are presented as mean ± standard deviation (SD) and compared using one-way analysis of variance (ANOVA). Categorical variables are summarized as frequencies and percentages, and were appropriately assessed using chi-square or Fisher’s exact tests.

To evaluate the independent associations of the AIP and its quartiles with SCH and thyroid hormone levels, weighted logistic and linear regression models were employed. A linear trend test was conducted by treating the median value of each AIP quartile as a continuous variable within the regression models. Model 1 was unadjusted. Model 2 was adjusted for demographic factors including gender, age, race, and educational level. On the basis of Model 2, Model 3 was further adjusted for lifestyle and clinical covariates such as smoking status, alcohol intake, BMI, urinary iodine concentration, and history of diabetes and cardiovascular disease.

To evaluate potential non-linear associations of the AIP with SCH and thyroid hormone levels, restricted cubic splines (RCS) were applied. Models with 3 to 7 knots were compared, and the optimal number of knots was selected based on the lowest Akaike Information Criterion (AIC), resulting in the use of 3 knots. The inflection point was identified by analyzing the fitted spline curve.

Mediation analysis was conducted using the “mediation” package in R (version 4.5.1) to evaluate the potential mediating role of TSH in the association between the AIP and SCH. The indirect effect of AIP mediated through TSH was compared with its total effect, with adjustment for gender, age, race, smoking status, alcohol consumption, BMI, urinary iodine levels, and history of diabetes mellitus and cardiovascular disease.

Subgroup analyses were performed using stratified logistic and linear regression models across prespecified strata, including age, sex, smoking status, BMI, diabetes, and cardiovascular disease. To evaluate potential effect modification and test the robustness of the primary findings, interaction effects between each covariate and the AIP were examined.

All animal experimental data are expressed as mean ± SD. Statistical analyses were conducted using SPSS version 26.0 (IBM Corp., USA). Differences between two groups were compared using unpaired Student’s t-test. Given the sample size, Pearson correlation analyses between AIP and thyroid parameters (TSH, FT3, FT4) within each group were considered exploratory, aimed at generating hypotheses regarding their relationship under different metabolic conditions. A two-tailed P value less than 0.05 was considered statistically significant.

As this study is a secondary analysis of the pre-existing, nationally representative NHANES database and included all eligible participants from the 2007–2012 cycles, an *a priori* power calculation was not conducted. The analytical approach prioritizes the utilization of the entire available cohort to maximize representativeness and precision, which is a standard methodology for such survey-based analyses.

## 3 Results

### 3.1 Baseline characteristics by AIP quartiles

A total of 3,135 participants were enrolled in this study, consisting of 1,720 males and 1,415 females. According to quartiles of the AIP, the participants were categorized into four groups (Q1 - Q4). Compared to those in the lowest AIP quartile (Q1), subjects with higher AIP values were more likely to be male, of non-Hispanic White, overweight or obese, and to have cardiovascular disease or diabetes. Furthermore, these individuals exhibited higher BMI, TC, TG, and LDL-C levels, along with lower HDL-C levels (all P < 0.05). The detailed baseline characteristics are summarized in **Table 1**.

**Table 1:** Baseline characteristics stratified by AIP quartiles.

Variables^1^	Total (n=3135)	AIP quartiles				Statistic	*P* value
		Q1 (n=784)	Q2 (n=783)	Q3 (n=784)	Q4 (n=784)		
Demographic Characteristics							
Age, years	49.16 ± 17.58	46.85 ± 17.84	48.88 ± 18.25	50.29 ± 17.59	51.62 ± 16.40	F=7.51	**<.001**
Age group, n(%)						χ²=24.48	**<.001**
18-44	1333 (42.52)	386 (49.05)	339 (43.35)	302 (38.97)	306 (38.69)		
45-59	733 (23.38)	156 (19.82)	175 (22.38)	194 (25.03)	208 (26.30)		
≥60	1069 (34.10)	245 (31.13)	268 (34.27)	279 (36.00)	277 (35.02)		
Gender, n(%)						χ²=118.07	**<.001**
Male	1720 (54.86)	325 (41.30)	401 (51.28)	463 (59.74)	531 (67.13)		
Female	1415 (45.14)	462 (58.70)	381 (48.72)	312 (40.26)	260 (32.87)		
Education level, n(%)						χ²=92.69	**<.001**
Less Than 9th Grade	374 (11.91)	55 (14.71)	94 (25.13)	85 (22.73)	140 (37.43)		
9-11th Grade	538 (17.16)	129 (23.98)	115 (21.38)	143 (26.58)	151 (28.07)		
High School graduate	730 (23.29)	157 (21.51)	188 (25.75)	196 (26.85)	189 (25.89)		
Some college	1491 (47.56)	446 (29.91)	384 (25.75)	351(23.54)	310 (20.79)		
Race, n(%)						χ²=175.11	**<.001**
Mexican American	507 (16.17)	70 (8.89)	132 (16.88)	144 (18.58)	161 (20.35)		
Other Hispanic	364 (11.61)	70 (8.89)	81 (10.36)	99 (12.77)	114 (14.41)		
Non-Hispanic White	1418 (45.23)	323 (41.04)	357 (45.65)	344 (44.39)	394 (49.81)		
Non-Hispanic Black	647 (20.64)	270 (34.31)	160 (20.46)	136 (17.55)	81 (10.24)		
Other Race	199 (6.35)	54 (6.86)	52 (6.65)	52 (6.71)	41 (5.18)		
Lifestyle Factors							
Alcohol intake, n(%)						χ²=38.46	**<.001**
Non-drinker	837 (26.70)	214 (27.19)	236 (30.18)	180 (23.23)	207 (26.17)		
1–4 drinks/month	1557 (49.67)	346 (43.96)	377 (48.21)	403 (52.00)	431 (54.49)		
5–9 drinks/month	246 (7.85)	65 (8.26)	55 (7.03)	69 (8.90)	57 (7.21)		
10+ drinks/month	495 (15.79)	162 (20.58)	114 (14.58)	123 (15.87)	96 (12.14)		
Smoking status, n(%)						χ²=75.22	**<.001**
Never smoker	1639 (52.28)	498 (63.28)	430 (54.99)	366 (47.23)	345 (43.62)		
Former smoker	711 (22.68)	139 (17.66)	165 (21.10)	183 (23.61)	224 (28.32)		
Current smoker	785 (25.04)	150 (19.06)	187 (23.91)	226 (29.16)	222 (28.07)		
Anthropometric Measures							
BMI,kg/m^2^	28.64 ± 6.37	26.01 ± 5.97	28.18 ± 6.22	29.74 ± 6.30	30.62 ± 6.02	F=85.30	**<.001**
BMI group, n(%)						χ²=290.13	**<.001**
Normal weight	949 (30.27)	395 (50.19)	265 (33.89)	169 (21.81)	120 (15.17)		
Overweight	1097 (34.99)	242 (30.75)	265 (33.89)	287 (37.03)	303 (38.31)		
Obesity	1089 (34.74)	150 (19.06)	252 (32.23)	319 (41.16)	368 (46.52)		
Biochemical Data							
FT4,ng/dl	0.80 ± 0.14	0.81 ± 0.13	0.81 ± 0.14	0.80 ± 0.14	0.79 ± 0.14	F=3.34	**0.019**
TT4, ug/dl	7.93 ± 1.54	7.69 ± 1.52	7.97 ± 1.55	8.04 ± 1.56	8.02 ± 1.53	F=8.76	**<.001**
FT3, pg/ml	3.24 ± 0.60	3.16 ± 0.36	3.26 ± 1.00	3.25 ± 0.40	3.30 ± 0.36	F=8.20	**<.001**
TT3, ng/dl	115.86 ± 23.15	110.72 ± 22.06	115.42 ± 22.35	117.72 ± 23.88	119.60 ± 23.37	F=22.00	**<.001**
Tg, ng/ml	13.79 ± 2.26	14.26 ± 1.01	13.49 ± 2.26	13.97 ± 3.02	13.45 ± 1.74	F=0.80	0.495
TSH, mIU/L	1.89 ± 0.13	1.73 ± 0.02	1.82 ± 0.09	1.96 ± 0.21	2.04 ± 0.18	F=11.96	**<.001**
TG, mmol/L	1.40 ± 0.75	0.70 ± 0.21	1.06 ± 0.23	1.46 ± 0.29	2.39 ± 0.67	F=2616.08	**<.001**
TC,mg/dl	193.84 ± 40.34	186.95 ± 37.17	189.60 ± 39.13	193.86 ± 39.92	204.88 ± 42.65	F=31.14	**<.001**
LDL-C, mg/dl	115.63 ± 35.35	105.41 ± 30.30	115.31 ± 33.83	119.89 ± 35.89	121.94 ± 38.58	F=35.21	**<.001**
HDL-C, mmol/L	1.38 ± 0.40	1.79 ± 0.40	1.44 ± 0.28	1.24 ± 0.22	1.05 ± 0.21	F=941.48	**<.001**
UIC, ug/L	124.80 ± 24.30	121.44 ± 17.00	121.14 ± 21.25	126.49 ± 24.81	130.10 ± 23.80	F=2.65	**0.047**
Clinical History							
Presence of subclinical hypothyroidism, n(%)						χ²=9.77	**0.021**
No	3031 (96.68)	773 (98.22)	756 (96.68)	747 (96.39)	755 (95.45)		
Yes	104 (3.32)	14 (1.78)	26 (3.32)	28 (3.61)	36 (4.55)		
Presence of diabetes, n(%)						χ²=55.31	**<.001**
No	2768 (88.29)	731 (92.88)	713 (91.18)	677 (87.35)	647 (81.80)		
Yes	367 (11.71)	56 (7.12)	69 (8.82)	98 (12.65)	144 (18.20)		
Presence of cardiovascular disease, n(%)						χ²=29.54	**<.001**
No	2806 (89.51)	732 (93.01)	713 (91.18)	688 (88.77)	673 (85.08)		
Yes	329 (10.49)	55 (6.99)	69 (8.82)	87 (11.23)	118 (14.92)		

^1^Normally distributed continuous variables were presented as the mean ± standard deviation, and group comparisons were performed using one-way analysis of variance (ANOVA); Categorical variables were presented as frequencies and percentages [n (%)], and group comparisons were performed using the chi-square test or Fisher’s exact test.

AIP, atherogenic index of plasma; FT3, free triiodothyronine; FT4, free thyroxine; TSH, thyroid-stimulating hormone; TT3, total triiodothyronine; TT4, total thyroxine; Tg, thyroglobulin; BMI, body mass index; TG: triglycerides, TC: total cholesterol, HDL-C: high-density lipoprotein cholesterol, LDL-C: low-density lipoprotein cholesterol; UIC, urinary iodine concentration, Q, quartile.Bold values indicate statistical significance at P < 0.05.

### 3.2 The association between AIP and SCH

**Figure 2:**
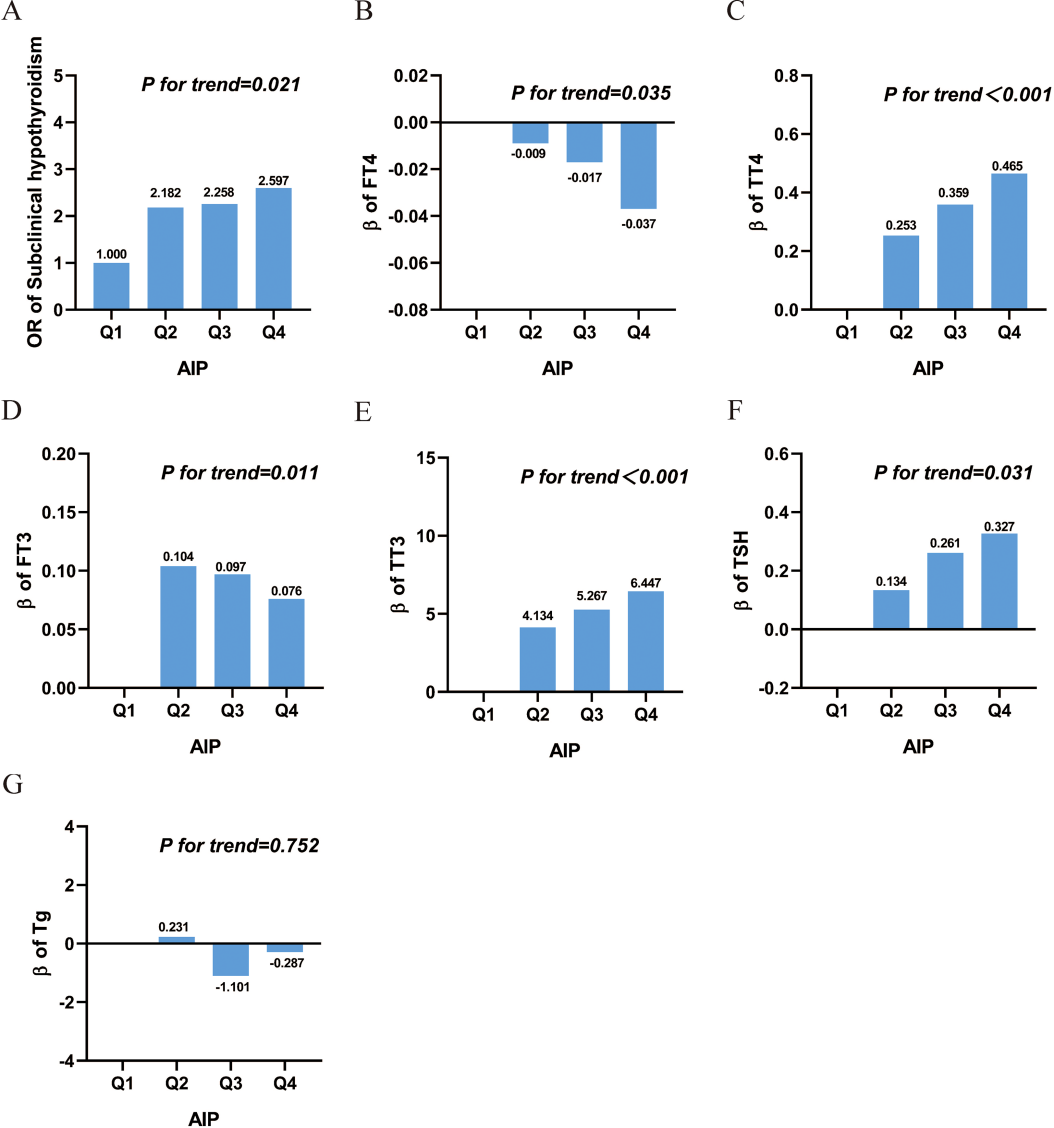
Association between AIP and SCH and thyroid hormone. **(A)** Subclinical hypothyroidism; **(B)** free thyroxine; **(C)** total thyroxine; **(D)** free triiodothyronine; **(E)** total Triiodothyronine; **(F)** thyroid-stimulating hormone; **(G)** thyroglobulin. Abbreviations: AIP, atherogenic index of plasma; Q, quartile; FT3, free triiodothyronine; FT4, free thyroxine; TSH, thyroid-stimulating hormone; TT3, total triiodothyronine; TT4, total thyroxine; Tg, thyroglobulin; OR: Odds Ratio.

AIP was significantly associated with an increased risk of SCH, both as a continuous variable and quartiles. In the fully adjusted model (Model 3), an increase in AIP was associated with a significantly elevated risk of SCH. Similarly, participants in the highest AIP quartile (Q4) had substantially greater odds of SCH compared to those in the lowest quartile (Q1) (OR = 2.597, 95% CI: 1.657 - 4.412; P for trend = 0.021), as shown in **Figure 2A** and **Table 2**.

**Table 2:** Association between AIP and SCH.

	Participants	SCH	Model1^a^	P value	Model 2^b^	P value	Model 3^c^	P value
			OR (95% CI)		OR (95% CI)		OR (95% CI)	
AIP	3135	104	2.262 (1.237, 3.176)	0.011	2.412 (1.194, 4.164)	0.016	2.417 (1.239, 4.214)	0.014
quartile								
Q1	784	14	—		—		—	
Q2	783	26	2.135 (0.964, 3.244)	0.093	2.143 (0.987, 3.157)	0.073	2.182 (0.984, 4.697)	0.072
Q3	784	28	1.994 (0.963, 3.818)	0.071	1.941 (1.000, 3.842)	0.057	2.258 (1.147, 4.563)	0.014
Q4	784	36	2.313 (1.369, 3.872)	0.016	2.264 (1.324, 3.866)	0.014	2.597 (1.657, 4.412)	0.016
P for trend			0.024		0.017		0.021	

AIP, atherogenic index of plasma; SCH, Subclinical Hypothyroidism; Q, quartile; OR, Odds Ratio; CI, Confidence Interval。

Weighted logistic regression was used to evaluate the association between AIP and SCH.

a Model 1 was not adjusted.

b Model 2 was adjusted for age, gender, race, and education.

c Model 3 was adjusted for age, gender, race, education, alcohol intake, smoking status, BMI, diabetes, cardiovascular disease, and urinary iodine concentratione.

### 3.3 The association between AIP and serum thyroid function indicators

After full adjustment for covariates, elevated AIP was significantly associated with a detrimental thyroid hormone profile: it correlated negatively with FT4 levels, and positively with TT4, FT3, TT3, and TSH (all P < 0.05). When analyzed by quartiles, these associations remained statistically significant and exhibited a clear dose-response relationship, as evidenced by significant P values for trend (all < 0.05), as detailed in **Table 3** and **Figures 2B–F**. However, there was no significant association between AIP and TG, as shown in **Table 3** and **Figure 2G**.

**Table 3:** Association between AIP and Thyroid hormone.

	Model 1^a^	P value	Model 2^b^	P value	Model 3^c^	P value
	β (95% CI)		β (95% CI)		β (95% CI)	
FT4,ng/dl						
AIP	-0.023 (-0.034, 0.011)	0.172	-0.036 (-0.067, -0.015)	0.024	-0.042 (-0.071, -0.011)	0.027
quartile						
Q1	—		—		—	
Q2	0.002 (-0.017, 0.014)	0.845	-0.011 (-0.027, 0.009)	0.793	-0.009 (-0.024, 0.019)	0.687
Q3	-0.008 (-0.015, 0.009)	0.257	-0.016 (-0.034, -0.009)	0.039	-0.017 (-0.034, 0.011)	0.154
Q4	-0.013 (-0.028, 0.011)	0.211	-0.036 (-0.058, -0.012)	0.036	-0.037 (-0.061, -0.012)	0.037
P for trend	0.142		0.027		0.035	
TT4, ug/dl						
AIP	0.422 (0.183, 0.594)	< 0.001	0.612 (0.361, 0.874)	< 0.001	0.462 (0.171, 0.758)	0.012
quartile						
Q1	—		—		—	
Q2	0.257 (0.124, 0.431)	0.024	0.321 (0.132, 0.541)	0.011	0.253 (0.054, 0.467)	0.031
Q3	0.357 (0.168, 0.514)	0.001	0.476 (0.269, 0.687)	< 0.001	0.359 (0.183, 0.612)	0.009
Q4	0.374 (0.214, 0.565)	< 0.001	0.512 (0.334, 0.674)	< 0.001	0.465 (0.286, 0.567)	0.014
P for trend	< 0.001		< 0.001		< 0.001	
FT3, pg/ml						
AIP	0.184 (0.097, 0.275)	< 0.001	0.126 (0.074, 0.179)	< 0.001	0.121 (0.067, 0.172)	0.017
quartile						
Q1	—		—		—	
Q2	0.142 (0.087, 0.241)	0.007	0.112 (0.045, 0.179)	0.003	0.104 (0.045, 0.172)	0.011
Q3	0.114 (0.096, 0.229)	< 0.001	0.093 (0.047, 0.123)	0.001	0.097 (0.037, 0.143)	0.021
Q4	0.154 (0.114 ,0.213)	< 0.001	0.109 (0.076, 0.145)	< 0.001	0.076 (0.056, 0.147)	< 0.001
P for trend	< 0.001		< 0.001		0.011	
TT3, ng/dl						
AIP	10.174 (6.124, 14.211)	< 0.001	10.036 (6.041, 14.039)	< 0.001	8.069 (3.799, 12.376)	0.013
quartile						
Q1	—		—		—	
Q2	5.311 (2.567, 7.124)	< 0.001	5.261 (2.876, 7.667)	< 0.001	4.134 (1.654, 6.635)	0.023
Q3	7.113 (3.342, 10.875)	< 0.001	7.178 (3.496, 10.857)	< 0.001	5.267 (2.186, 9.746)	0.015
Q4	8.247 (5.127, 11.413)	< 0.001	8.161 (5.137, 11.174)	< 0.001	6.447 (3.267, 9.586)	< 0.001
P for trend	< 0.001		< 0.001		< 0.001	
TSH, mIU/L						
AIP	0.355 (0.142, 0.511)	0.001	0.402 (0.173, 0.621)	0.001	0.425 (0.186, 0.687)	0.011
quartile						
Q1	—		—		—	
Q2	0.131 (-0.043, 0.264)	0.121	0.122 (-0.064, 0.291)	0.174	0.134 (-0.051, 0.296)	0.156
Q3	0.241 (0.069, 0.386)	0.023	0.232 (0.099, 0.387)	0.011	0.261 (0.134, 0.431)	0.014
Q4	0.289 (0.131, 0.425)	0.001	0.312 (0.177, 0.484)	0.013	0.327 (0.153, 0.492)	0.024
P for trend	0.001		0.021		0.031	
Tg, ng/ml						
AIP	-0.061 (-2.695, 2.721)	0.914	2.826 (-0.374, 5.998)	0.094	0.251 (-3.293, 3.781)	0.745
quartile						
Q1	—		—		—	
Q2	0.174 (-3.597, 4.065)	0.817	1.226 (-2.764, 5.192)	0.621	0.231 (-3.411, 3.634)	0.846
Q3	-1.265 (-3.398, 1.036)	0.396	0.461 (-1.874, 2.769)	0.813	-1.101 (-3.864, 1.672)	0.421
Q4	-0.164 (-2.513, 2.231)	0.754	1.916 (-0.776, 4.587)	0.197	-0.287 (-3.356, 2.791)	0.875
P for trend	0.599		0.285		0.752	

AIP, atherogenic index of plasma; Q, quartile; FT3, free triiodothyronine; FT4, free thyroxine; TSH, thyroid-stimulating hormone; TT3, total triiodothyronine ; TT4, total thyroxine; Tg, thyroglobulin.

Weighted regression analysis was used to evaluate the relationship between AIP and thyroid hormone.

a Model 1 was not adjusted.

b Model 2 was adjusted for age, gender, race, and education.

c Model 3 was adjusted for age, gender, race, education, alcohol intake, smoking status, BMI, diabetes, cardiovascular disease, and urinary iodine concentratione.

### 3.4 RCS analysis investigating potential nonlinear relationship between AIP and SCH and serum thyroid function indicators

**Figure 3:**
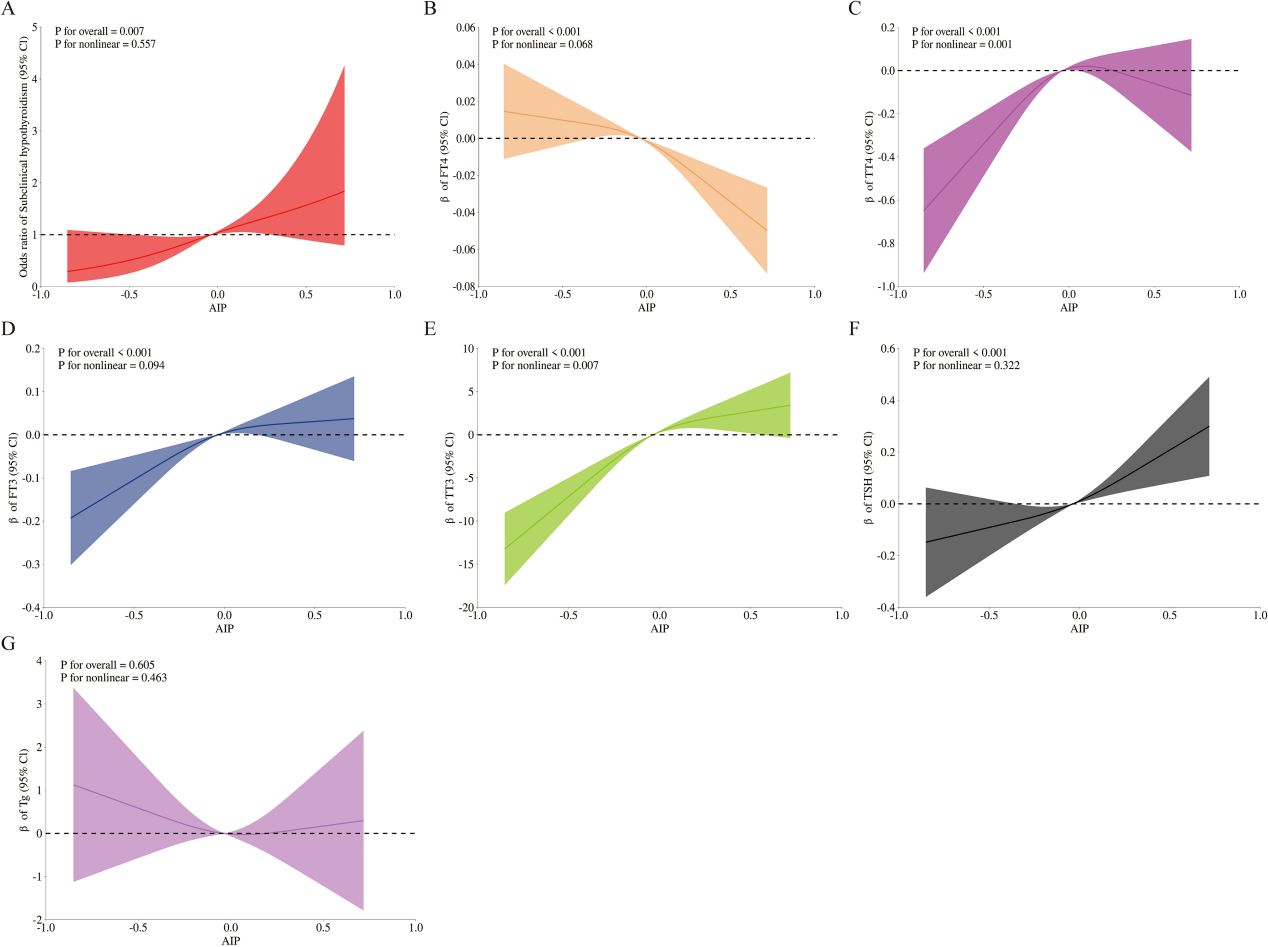
Smooth curve fitting of AIP with SCH and thyroid hormone. Solid lines represent the smooth curve fit between variables, and shaded bands indicate the 95% confidence intervals (CIs) for the fit. **(A)** Subclinical hypothyroidism; **(B)** FT4; **(C)** TT4; **(D)** FT3; **(E)** TT3; **(F)** TSH; **(G)** Tg. All graphs were adjusted for age, gender, race, education, alcohol intake, smoking status, BMI, diabetes, cardiovascular disease, and urinary iodine concentratione. Abbreviations: AIP, atherogenic index of plasma; Q, quartile; FT3, free triiodothyronine; FT4, free thyroxine; TSH, thyroid-stimulating hormone; TT3, total triiodothyronine; TT4, total thyroxine; Tg, thyroglobulin.

Restricted cubic splines were used to flexibly model and visualize the associations of AIP with SCH and thyroid function parameters, as presented in **Figure 3**. After full adjustment for covariates, linear associations were identified between AIP and SCH, FT4, FT3, and TSH (P for overall < 0.05; P for nonlinear > 0.05). AIP exhibited nonlinear relationships with both TT3 and TT4 (P for overall < 0.05; P for nonlinear < 0.05).

### 3.5 The role of TSH in the association between AIP and SCH: a mediation analysis

**Figure 4:**
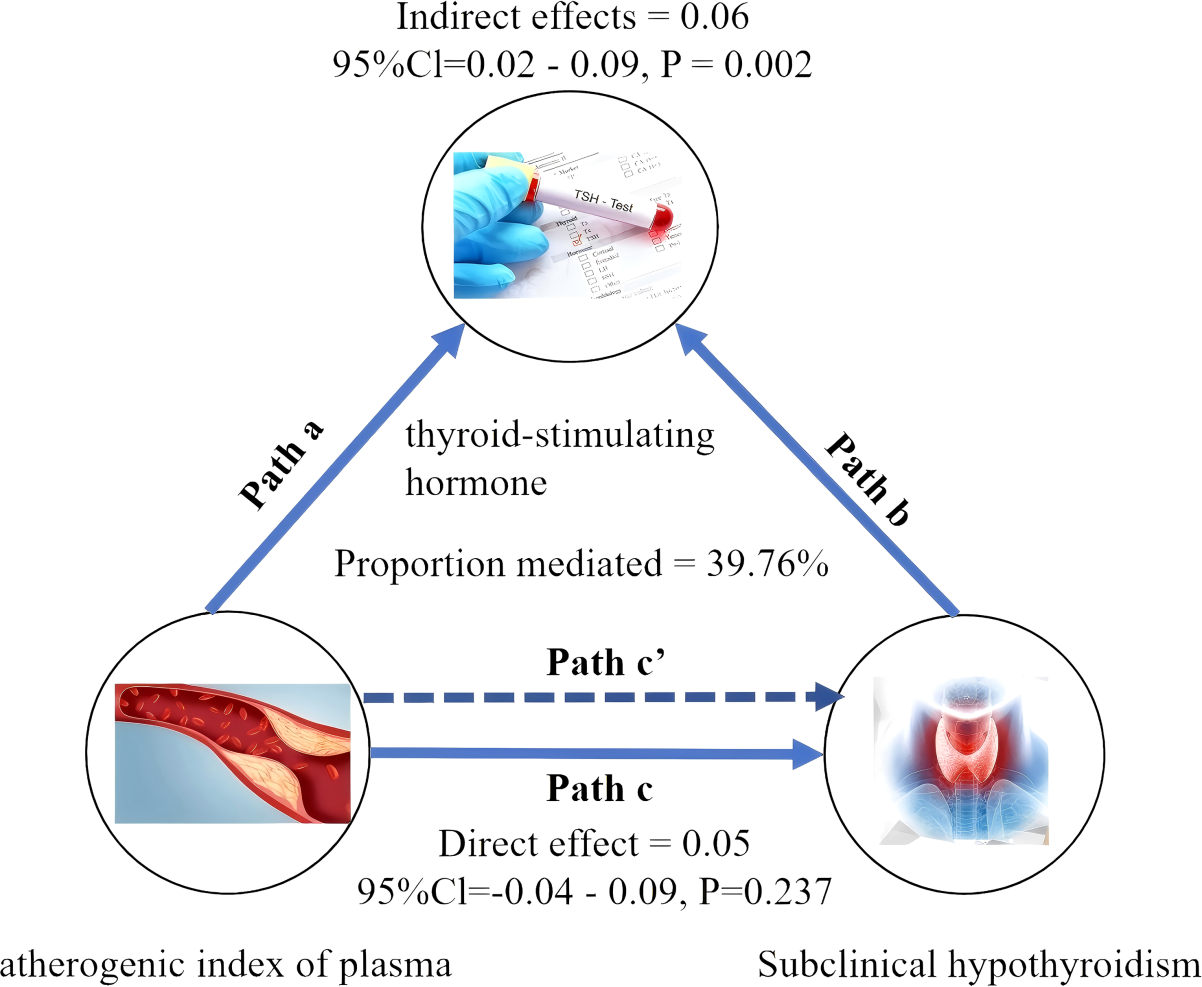
Mediated analysis model path diagram. atherogenic index of plasma was defined as the independent variable; subclinical hypothyroidism as the dependent variable; and thyroid-stimulating hormone as the mediating variable. Path a represents the regression coefficient of the association between atherogenic index of plasma and thyroid-stimulating hormone. Path b represents the regression coefficient of the association between thyroid-stimulating hormone and subclinical hypothyroidism. Path c represents the simple total effect of atherogenic index of plasmaon on subclinical hypothyroidism. Path c’ represents the direct effect of atherogenic index of plasmaon on subclinical hypothyroidism.

Mediation analysis was conducted to explore the potential mediating role of TSH in the relationship between AIP and SCH. **Figure 4** depicts the conceptual mediation model, where AIP is treated as the independent variable, TSH as the hypothesized mediator, and SCH as the dependent variable. The findings indicated a statistically significant indirect effect of AIP on SCH prevalence through TSH, with an indirect effect size of 0.06 (95% CI: 0.02 - 0.09; P = 0.002). This suggests that TSH may statistically explain a part of the observed association between AIP and SCH, with a proportion of 39.76% of the total association accounted for by this pathway, as shown in **Table 4**.

**Table 4:** Analysis of the mediating role of TSH in the association between AIP and SCH.

Independent variables	Mediating variables	Sample	Total effect		Direct effect		Indirect effects		Proportion of mediators, %
			Coefficient (95% CI)	P value	Coefficient (95% CI)	P value	Coefficient (95% CI)	P value	
AIP	TSH	3135	0.09 (0.04 - 0.13)	0.024	0.05 (-0.04 - 0.09)	0.237	0.06 (0.02 - 0.09)	0.002	39.76%

AIP, atherogenic index of plasma; TSH, thyroid-stimulating hormone; SCH, Subclinical hypothyroidism.

The model was adjusted for age, gender, race, education, alcohol intake, smoking status, BMI, diabetes, cardiovascular disease, and urinary iodine concentratione.

### 3.6 Subgroup analysis

**Supplementary Materials 1–2** present subgroup analyses stratified by sex, age, smoking status, BMI, diabetes, and cardiovascular disease, to investigate potential effect modifiers in the association between AIP and SCH as well as thyroid function parameters. The results indicated that the association between AIP and SCH, as well as thyroid function indicators, remained consistent across subgroups stratified by sex, age, smoking status, BMI, diabetes, and cardiovascular disease. None of the interaction terms between AIP and these covariates reached statistical significance (all P for interaction > 0.05).

### 3.7 Thyroid function alterations in SD rats on high-fat diet

**Figure 5:**
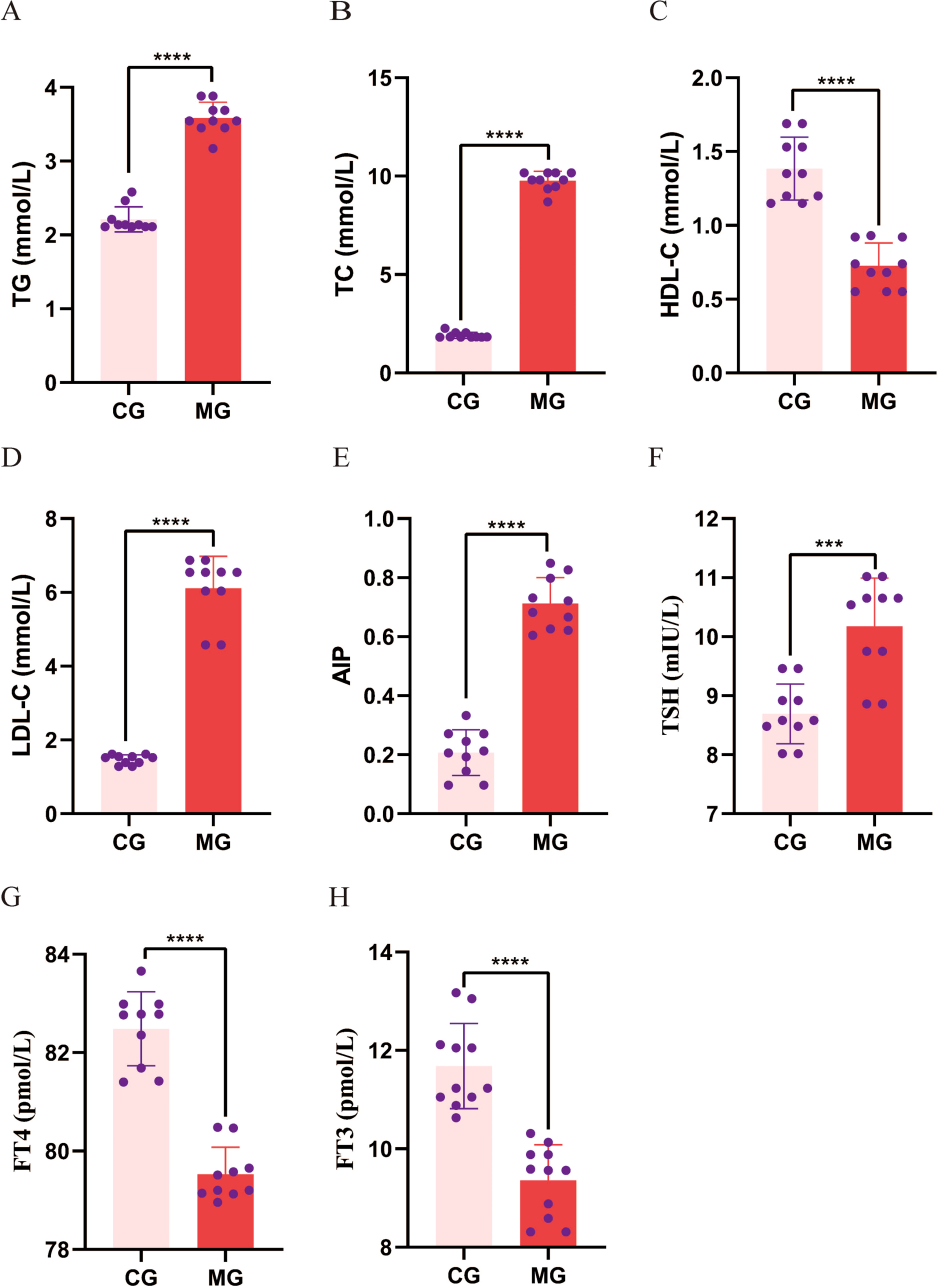
Comparison of blood lipid and thyroid function levels between control and model groups. **(A-D)** Serum TG, TC, HDL-C and LDL-C; **(E)** AIP calculated from serum TG and HDL-C; **(F-H)** Serum TSH, FT4 and FT3. Data are expressed as mean ± standard deviation. ***p < 0.001, *****p < 0.0001. N = 10 per group. CG, Control group; MG, Model group; TG, triglycerides; TC, total cholesterol, HDL, high-density lipoprotein cholesterol; LDL, low-density lipoprotein cholesterol; AIP, atherogenic index of plasma; TSH, thyroid-stimulating hormone; FT4, free thyroxine; FT3, free triiodothyronine.

As presented in **Figure 5**, compared with the CG, the MG exhibited significantly elevated serum levels of TC, TG, and LDL-C, alongside a significant reduction in HDL-C, indicating disrupted lipid metabolism. The MG exhibited significantly higher AIP values than the CG. Relative to the CG, the MG demonstrated a significant increase in serum TSH and decreases in FT4 and FT3.

**Figure 6:**
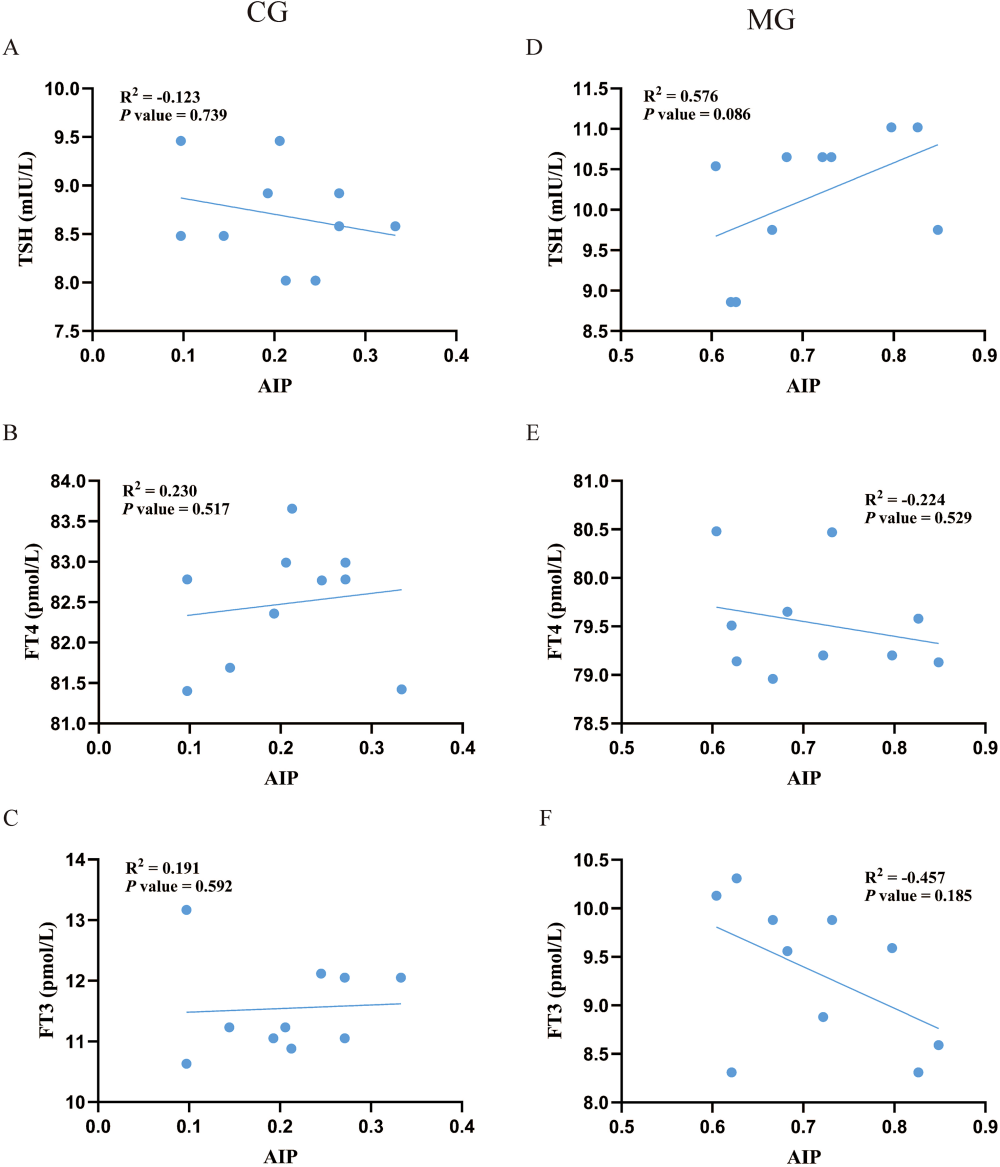
Pearson correlation analysis between AIP with thyroid function. N = 10 per group. **(A-C)** correlation analysis of AIP with serum TSH, FT3 and FT4 in the CG. **(D-F)** correlation analysis of AIP with serum TSH, FT3 and FT4 in the MG. CG, Control group; MG, Model group;AIP, atherogenic index of plasma; TSH, thyroid-stimulating hormone; FT4, free thyroxine; FT3, free triiodothyronine.

Exploratory analysis of the association between AIP and thyroid parameters within each group revealed an intriguing pattern (**Figure 6**). In the CG, AIP showed non-significant trends of a negative correlation with TSH and positive correlations with FT3 and FT4. Conversely, in the MG, these relationships were reversed, with AIP demonstrating non-significant trends of a positive correlation with TSH and negative correlations with FT3 and FT4. Although these correlations did not reach statistical significance, the opposing directions of the trends suggest that the physiological interplay between AIP and thyroid function may differ between normal and dyslipidemic states.

## 4 Discussion

In recent decades, cross-sectional studies have indicated a link between SCH and dyslipidemia (21–23). However, these investigations have primarily considered dyslipidemia as an outcome of SCH. With advancing understanding of the condition, emerging evidence proposes that dyslipidemia may also act as a risk factor for SCH. A Korean cohort study demonstrated that dyslipidemia significantly increases the risk of developing SCH (24). Furthermore, a retrospective cohort analysis using data from the REACTION study revealed a significant association between dynamic changes in lipid profiles and the presence of SCH (25). Conventional single lipid parameters offer limited insights into the complex dyslipidemia associated with SCH, whereas novel composite lipid indices provide a more holistic assessment of lipid metabolism than individual lipids. Therefore, investigating the relationship between novel composite lipid indices and SCH is of considerable importance. Notably, AIP has been established as a predictive marker for disordered lipid metabolism. Nonetheless, there remains a scarcity of literature examining the correlation between AIP and SCH, and the clinical relevance of AIP in the context of SCH has yet to be elucidated.

This study is among the first to provide epidemiological evidence that AIP is significantly associated with SCH and a spectrum of thyroid function parameters. Crucially, these associations persisted after fully adjustment for confounders and were robust across subgroup analyses. The identification of both linear and specific nonlinear relationships (particularly the inverted U-shaped association with TT4) suggests that the association between dyslipidemia and thyroid physiology may involve complex, threshold-dependent mechanisms. Furthermore, our mediation analysis indicated that TSH was identified as an explanatory factor, accounting for 39.76% of the association between AIP and SCH. Collectively, these findings reinforce a close link between lipid metabolism and thyroid function.

To substantiate these epidemiological observations, we employed a high-fat diet rat model. The animal experiment successfully recapitulated the core human finding: induction of dyslipidemia (evidenced by elevated AIP) coincided with elevated TSH and reduced FT4 and FT3. This aligns with previous studies linking high-fat diets to thyroid hormonal changes (26–28). Beyond this confirmation, the controlled experimental setting revealed an additional layer of complexity: the exploratory analysis suggested that the correlative patterns between AIP and thyroid hormones appeared to differ between dyslipidemic and normal metabolic states. This nuanced finding, which is uniquely accessible through an experimental model, indicates that the relationship between lipid metabolism and thyroid function may be context-dependent. It thereby generates novel hypotheses for future research regarding the physiological interplay between these systems.

To our knowledge, this study is among the first to investigate the relationship between AIP and both SCH and thyroid function. Previous research has primarily focused on the associations between individual lipid markers and thyroid dysfunction. For instance, data from a study in Taiwan, China, indicated that elevated TG levels are associated with an increased risk of SCH (29). A two-sample bidirectional Mendelian randomization analysis revealed that genetically predicted HDL-C was inversely associated with TSH levels (30). Additionally, a cross-sectional study conducted in Iran reported that individuals with dyslipidemia had significantly lower FT4 and higher TSH levels compared to those without dyslipidemia (31). Despite these findings, no study to date has specifically examined the effect of AIP on SCH and thyroid function. AIP—a readily available composite metric derived from TG and HDL-C—provides a more comprehensive reflection of lipid-related pathogenicity than individual lipid parameters (32). The relationship between AIP and both SCH and thyroid function remains unclear and has not been thoroughly established in existing literature. Our findings address this void by demonstrating that higher AIP levels are positively correlated with SCH and TSH, and negatively correlated with FT4, which aligns with and extends aforementioned findings on dyslipidemia and thyroid dysfunction.

Potential pathways connecting AIP to thyroid function, inferred from the existing literature, may involve disrupted adipokine signaling, chronic inflammation, suppressed deiodinase activity, and oxidative stress. Central to this may be the disruption of the hypothalamic-pituitary-thyroid (HPT) axis, potentially driven by AIP-altered adipokine signaling (e.g., leptin) (33), which can stimulate TSH secretion. Concurrently, the pro-inflammatory state fostered by dyslipidemia can directly suppress thyroid peroxidase activity and thyrocyte function, impairing hormone synthesis (11, 34). Furthermore, peripheral thyroid hormone metabolism may be compromised by deiodinase activity (e.g., DIO2) suppressed as a consequence of dyslipidemia, which reduces the conversion of T4 to active T3 and creates a tissue-level hypothyroid state (12, 35). Finally, dyslipidemia-induced oxidative stress can inflict direct damage on thyrocytes, further disrupting hormonal homeostasis (36, 37). These pathways collectively contribute to the thyroid dysfunction observed in conjunction with a high AIP.

This study possesses several notable strengths. First, to our knowledge, it is among the first to systematically evaluate the association between AIP and SCH as well as thyroid hormones within a large, nationally representative sample, which enhances the generalizability and clinical relevance of the findings. Second, the combination of population-based observational data and animal experiments provides multi-level validation of the results, significantly strengthening the reliability and translational value of the conclusions.

Nevertheless, several limitations of this study should be acknowledged. First, The cross-sectional design of the NHANES-based analysis limits the ability to establish causal relationships between AIP and thyroid dysfunction. Second, thyroid function indicators were measured only at a single time point, which may not capture their natural variation or long-term dynamics. Third, the discussion on potential biological pathways is based on inferences from the established literature. Our study did not include direct measurements of molecular factors (e.g., specific inflammatory cytokines, leptin, or deiodinase activity). Therefore, while these pathways provide a plausible context for our findings, their specific roles in the observed association between AIP and thyroid function remain undefined within the scope of this study. Furthermore, the modest sample size limited the statistical power of the within-group correlation analyses; although intriguing divergent trends were observed, they did not reach statistical significance and should be considered exploratory and hypothesis-generating. Fourth, the use of self-reported data for conditions such as diabetes, cardiovascular disease, and alcohol intake is a limitation, as it is prone to recall bias and potential misclassification. Finally, although we adjusted for the history of diabetes and cardiovascular disease as covariates, we cannot rule out residual confounding by medications used to treat these conditions. These medications are known to alter lipid profiles (and thus AIP) and may also have pleiotropic effects on thyroid function. Therefore, the observed associations should be interpreted with caution in this context. Future studies with detailed medication information are warranted to clarify this potential confounding.

In conclusion, this cross-sectional study demonstrates a significant association between elevated AIP and a higher prevalence of SCH, with TSH being an interrelated factor. Animal experiments provided supportive evidence, showing that high AIP levels co-occur with elevated TSH and reduced FT3 and FT4 levels. Collectively, these findings from human and animal data highlight an association between AIP and thyroid dysfunction parameters. It is crucial to emphasize that the cross-sectional design of the primary analysis does not allow for causal inference regarding the direction of this relationship. Future longitudinal studies are needed to clarify the temporal and causal pathways between AIP and thyroid function.

## Data Availability

Publicly available datasets were analyzed in this study. The data in the current study can be found on the website: https://wwwn.cdc.gov/nchs/nhanes/.
