# Supplementary material for: Relationship between plasma atherogenic index and subclinical hypothyroidism: an analysis of NHANES data and animal experiment
Source: Front Endocrinol (Lausanne). 2025 Nov 3;16:1700853. doi: 10.3389/fendo.2025.1700853 (PMC12621276; doi:10.3389/fendo.2025.1700853)
Supplement: Supplementary file 2 [file Table2.docx]

**Supplementary Material 2 Subgroup Analysis of the Association between AIP and FT3, TT3, TSH and Tg**

| Subgroup | FT3 |  | TT3 |  | TSH | Tg |
| --- | --- | --- | --- | --- | --- | --- |
|  | OR(95%CI) Pvalue |  | OR (95%CI) Pvalue |  | OR(95%CI) Pvalue | OR(95%CI) Pvalue |
| Gender |  |  |  |  |  |  |
| Male | 0.247 (0.154, 0.398) 0.025 |  | 6.425 (2.635, 10.963) 0.032 |  | 0.588 (0.356, 1.098) 0.036 | -2.653 (-8.452, 4.326) 0.263 |
| Female | 0.112 (-0.024, 0.103) 0.164 |  | 10.623 (6.275, 15.243) 0.026 |  | 0.263 (-0.123, 0.416) 0.265 | -0.975 (-2.653, 3.627) 0.423 |
| P for interaction | 0.544 |  | 0.236 |  | 0.096 | 0.826 |
| Age |  |  |  |  |  |  |
| 20-44 | 0.045 (-0.024, 0.078) 0.174 |  | 7.321 (3.287, 12.423) 0.013 |  | 0.152 (-0.096, 0.275) 0.102 | 2.635 (-2.165,7.633) 0.632 |
| 45-59 | 0.285 (0.109, 0.398) 0.041 |  | 7.256 (1.365, 10.745) 0.012 |  | 0.324 (-0.015,0.654) 0.165 | 2.654 (-1.657, 6.345) 0.598 |
| ≥60 | 0.089 (-0.097, 0.145) 0.365 |  | 9.632 (4.256, 14.462) 0.026 |  | 0.356 (-0.096, 0.489) 0.096 | -1.649 (-8.746, 1.362) 0.165 |
| P for interaction | 0.745 |  | 0.625 |  | 0.765 | 0.189 |
| Smoking status |  |  |  |  |  |  |
| Never smoker | 0.074 (-0.045, 0.121) 0.213 |  | 8.534 (4.614, 12.421) 0.024 |  | 0.256 (0.096, 0.587) 0.029 | -1.632 (-5.648,3.698) 0.265 |
| Former smoker | 0.054 (-0.042, 0.103) 0.245 |  | 4.265 (-2.254, 6.547) 0.369 |  | 0.574 (0.174, 1.097) 0.046 | 2.148 (-1.659, 8.623) 0.365 |
| Current smoker | 0.198 (0.099, 0.354) 0.015 |  | 13.623 (7.245, 19.963) 0.029 |  | 0.412 (-0.152, 0.687) 0.154 | -2.645 (-6.356, 5.962) 0.465 |
| P for interaction | 0.635 |  | 0.365 |  | 0.485 | 0.623 |
| BMI |  |  |  |  |  |  |
| Normal weight | 0.054 (-0.057, 0.076) 0.145 |  | 11.654 (5.354, 17.687) 0.014 |  | 0.354 (0.102, 0.865) 0.024 | 0.623 (-2.631, 3.145) 0.346 |
| Overweight | 0.065 (-0.059, 0.098) 0.215 |  | 6.542 (1.934, 9.563) 0.036 |  | 0.384 (-0.099, 0.745) 0.127 | 2.984 (-1.362, 3.624) 0.362 |
| Obesity | 0.064 (-0.046, 0.121) 0.623 |  | 5.625 (2.958, 8.654) 0.013 |  | 0.374 (-0.069, 0.674) 0.108 | -2.351 (-3.627, 1.421) 0.237 |
| P for interaction | 0.475 |  | 0.211 |  | 0.635 | 0.269 |
| Diabetes |  |  |  |  |  |  |
| No | 0.156 (0.098, 0.254) 0.037 |  | 8.632 (5.853, 12.354) 0.029 |  | 0.354 (0.120, 0.589) 0.028 | -1.521 (-2.876, 2.954) 0.512 |
| Yes | 0.054 (-0.065, 0.102) 0.354 |  | 6.531 (-2.824, 10.743) 0.231 |  | 0.748 (0.295, 1.052) 0.018 | -0.984 (-1.426, 1.173) 0.843 |
| P for interaction | 0.846 |  | 0.423 |  | 0.231 | 0.714 |
| Cardiovascular |  |  |  |  |  |  |
| No | 0.164 (0.099,0.321) 0.014 |  | 7.524 (4.963, 11.785) 0.025 |  | 0.314 (0.109, 0.607) 0.026 | -2.623 (-5.14, 2.967) 0.274 |
| Yes | 0.065 (-0.035,0.112) 0.145 |  | 9.639 (-0.168, 15.154) 0.068 |  | 0.351 (-0.254, 0.962) 0.623 | -2.654 (-6.342, 2.721) 0.362 |
| P for interaction | 0.356 |  | 0.685 |  | 0.845 | 0.745 |
| Abbreviations：FT3，free triiodothyronine；TT3，total triiodothyronine；TSH，thyroid-stimulating hormone；Tg，thyroglobulin.  The model was adjusted for age, gender, race, educational, alcohol intake, smoking status, BMI, diabetes, cardiovascular disease, and urinary iodine concentration. In subgroup analyses, each stratification variable was excluded from the adjustment set in its respective subgroup model. | | | | | |  |
